# Stakeholder perceptions of components of a Parkinson disease care management intervention, care coordination for health promotion and activities in Parkinson’s disease (CHAPS)

**DOI:** 10.1186/s12883-020-02011-9

**Published:** 2020-12-02

**Authors:** Karen I. Connor, Hilary C. Siebens, Brian S. Mittman, Donna K. McNeese-Smith, David A. Ganz, Frances Barry, Lisa K. Edwards, Michael G. McGowan, Eric M. Cheng, Barbara G. Vickrey

**Affiliations:** 1 Veterans Affairs Parkinson’s Disease Research, Education and Clinical Center, Los Angeles, CA USA; 2 UCLA David Geffen School of Medicine, Los Angeles, CA USA; 3 Novato, CA USA; 4 Siebens Patient Care Communications LLC, Seal Beach, CA USA; 5 Kaiser Permanente Research, Pasadena, CA USA; 6 UCLA School of Nursing, Los Angeles, CA USA; 7 Veterans Affairs Geriatric Research, Education and Clinical Center and Center for the Study of Healthcare Innovation, Implementation and Policy, Los Angeles, CA USA; 8 Icahn School of Medicine at Mount Sinai, New York, NY USA

**Keywords:** Parkinson disease, Patient care management, Nursing process, Health communication, Case manager, Implementation, Dissemination

## Abstract

**Background:**

A recent nurse-led proactive care management intervention, Care Coordination for Health Promotion and Activities in Parkinson Disease (CHAPS), improved care quality when compared to usual care in a randomized controlled trial. Therefore, stakeholder (patient participants, nurse care managers, and Parkinson disease (PD) specialists) perceptions of key intervention components merit evaluation to inform decisions about dissemination.

**Methods:**

This multi-site study occurred in five southwest United States Veterans Health Administration medical centers. Stakeholders were surveyed on their perceptions of CHAPS including the CHAPS Assessment, CHAPS nurse care managers, the Siebens Domain Management Model™ (a practical clinical model), and the Siebens Health Care Notebook (Notebook) (self-care tool). Participants’ electronic medical records were abstracted for perceptions of the Notebook. Statistical analysis software was used to provide summary statistics; open card sorting methodology was used to identify themes and attributes in qualitative data including usability of some components.

**Results:**

Participants, overall, highly rated their medication self-management, acknowledged some challenges with the CHAPS self-care tools, reported knowledge of PD specialist follow-up and PD red flags, and rated CHAPS nurse care managers as helpful. Nurse care manager responses indicated the CHAPS Assessment and Program highly facilitated care of their patients. Most all PD specialists would refer other patients to CHAPS. Nurse care manager and PD specialist responses indicated improved participant management of their PD. Three themes emerged in participant perceptions of the Notebook: Notebook Assets (e.g., benefits and features-liked); Deferring Notebook Review (e.g., no time to review); and Reasons for Not Using (e.g., participant preference). Shared attributes regarding the Siebens Domain Management Model and Notebook usability, reported by nurse care managers, were user-friendly, person/patient-centered, and organized. Some challenges to their use were also reported.

**Conclusions:**

Overall, stakeholder perceptions of the proactive nurse-led CHAPS intervention indicated its value in the care of individuals with PD. Responses about the CHAPS Assessment, Siebens Domain Management Model, and Notebook self-care tool signified their usefulness. Stakeholders’ constructive suggestions indicated their engagement in CHAPS. These findings support CHAPS dissemination and contribute to research in care management.

**Trial registration:**

ClinicalTrials.gov as NCT01532986, registered on January 13, 2012.

**Supplementary Information:**

The online version contains supplementary material available at 10.1186/s12883-020-02011-9.

## Background

Parkinson’s disease (PD) care is evolving in response to the complexity of health-related problems that individuals experience [1, 2]. These efforts are timely as the incidence of PD will increase with population aging [3, 4]. Furthermore, an international emphasis on age-friendly care is ongoing [5, 6]. Recent studies have highlighted the importance of PD nurse specialists, as single point persons, to support patients and collaborate with PD specialists [1, 7]. However, a shortage of PD nurse specialists remains a barrier for many settings. Thus, a nurse-led care management intervention, Care Coordination for Health Promotion and Activities in Parkinson’s Disease (CHAPS), was designed to improve care quality through addressing the broad array of PD health issues [8].

The randomized controlled trial of the 18-month CHAPS intervention demonstrated improved adherence to PD quality indicators compared to usual care [9]. The trial was conducted between 2012 and 2017 at five Veterans Health Administration medical centers in the Southwest United States. These centers provide care to men and women who have served in the United States military. In the trial, community-dwelling patient/participants were the unit of randomization. Because the CHAPS intervention improved care quality, feedback from stakeholders (patient/participants, CHAPS nurse care managers, and PD specialists) is needed [10]. Direct participant feedback helps confirm if care was person/patient-centered [11–16]. Nurse care manager and PD specialist feedback provides insights on their engagement [17–19], likely representing buy-in required for adoption of new clinical approaches [20]. The purpose of this report is to describe stakeholder feedback on the CHAPS intervention and its components to inform decisions about CHAPS dissemination and contribute to research on improving PD care quality.

**Fig. 1:**
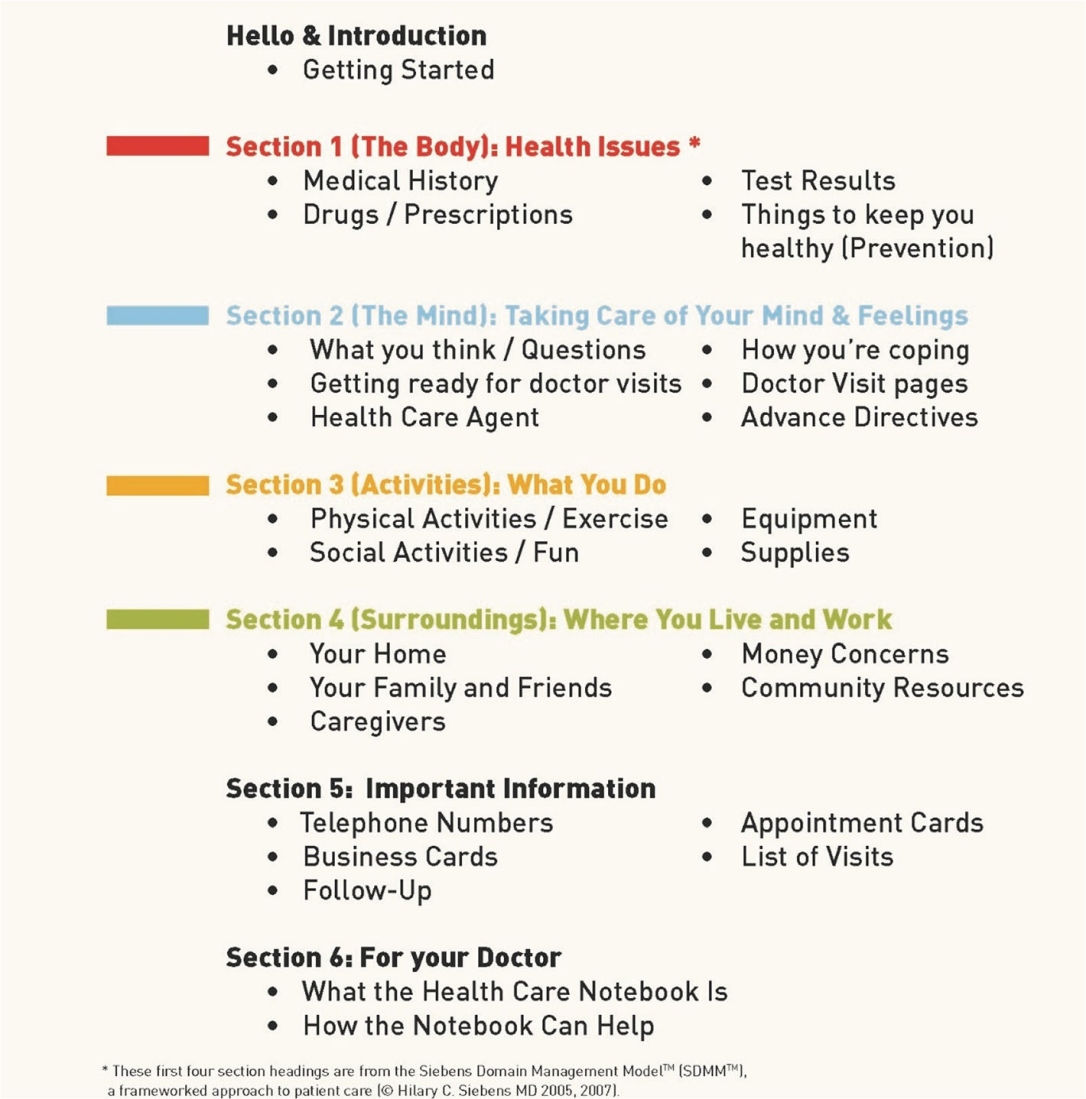
Section Contents of Self-care Tool: Siebens Health Care Notebook. LEGEND: Section contents of the Siebens Health Care Notebook (© 2008 Hilary C Siebens MD) [24]. The first four section headings are from the Siebens Domain Management Model™, an organizing framework for patient care

In brief, the CHAPS proactive care management intervention was led and provided by CHAPS nurse care managers oriented to PD care through a 10- to 40-h curriculum [8, 21–23]. These nurse care managers administered the same CHAPS Assessment to each participant for identifying problems/topics, and their severity, by embedded algorithms (Additional file 1) [8]. During follow-up calls, these nurse care managers could refer to problem/topic-specific intervention protocols for coaching participants in solving problems (e.g., medication, coping/self-management, falls, access to care and services). Self-care tools provided to participants included a self-care notebook (Fig. 1) [24] with a personalized action plan (Additional file 2) and a copy of their CHAPS Assessment results to share with others as desired [23].

Given the multiple ways PD affects day-to-day living, an organizing framework was necessary to help standardize and guide care management. The Siebens Domain Management Model™ [25, 26] was chosen as a synthesis of nursing [27, 28], biomedical, biopsychosocial [29], and biopsycho-ecological models [30]. It applies to any individual with any disease(s) or chronic/enduring health condition(s) in any care setting [8, 25] and has been shown to improve clinical outcomes [31–33]. This person/patient-centered care framework organizes individuals’ health-related strengths, problems, and topics into four orderly domains for following over time: I Medical/Surgical Issues, II Mental Status/Emotions/Coping, III Physical Function, and IV Living Environment (© Hilary C Siebens MD 2005) [23, 25, 31–37].

To use the four-domain concept with individuals and promote communication and self-management, each domain has a plain phrase name. These were determined with input from health literacy experts for corresponding sections in the Siebens Health Care Notebook (Notebook) [24, 25] (Fig. 1). This Notebook is a paper repository of health-related reminders and personalized education (after visit sheets, medication lists, education sheets, etc.). Notebooks have been used in randomized trials [38–40] and in quality improvement studies [41, 42] to assist self-care. Patient-held print records, like notebooks, have been noted as part of learning self-care in care transitions [43, 44] and care for enduring health conditions [45]. Recently, Notebook recipients treated for breast cancer, with difficulties in memory and thinking, endorsed it as a helpful tool in self-care [46].

We chose to assess usability of the Siebens Domain Management Model and the Notebook to inform decisions on dissemination. Usability testing had been helpful in finalizing the CHAPS Assessment in a previous pilot study, funded by Veterans Affairs Health Services Research and Development-Nurse Research Initiative (2008–2010). A research assistant had administered a 10-item usability survey about the drafted CHAPS Assessment to a convenience sample of 7 (28%) of 25 participants (unpublished KIC). The Assessment was found to be comprehensive, informative, and brought problems and topics to light that needed consideration, and question content was considered appropriate and not difficult. Some sensitive sections (e.g., incontinence, sexuality) required more explanation before questions were asked; thus, scripts for these sections were modified and the CHAPS Assessment was finalized.

## Methods

Aims were to evaluate: (1) participants’ knowledge of PD self-care and helpfulness of nurse care managers; (2) participants’ perceptions of the CHAPS Assessment; (3) participants’ responses to the health care Notebook; (4) CHAPS nurse care manager and PD specialist knowledge, beliefs, and attitudes about CHAPS and their perceptions of participants’ self-management; and (5) the usability of the Siebens Domain Management Model and the Notebook from the nurse care managers’ perspective.

### Setting and eligible participants

This study was conducted within the intervention arm of the CHAPS trial [9]. A total of 140 intervention participants received care management over an 18-month period. CHAPS nurse care manager staffing was about 125 participants per one full time employee equivalent. Routine assessments were the CHAPS Assessment, 6-month follow-ups, and annual reassessments. All participants received the CHAPS Assessment, averaging 120 min (standard deviation (SD) 78) requiring 2.1 encounters (SD 1.6, median 2.0) (i.e., participant/nurse care manager interactions). Annual reassessments (*n* = 29), designed to be briefer, averaged 32 min (SD 34) requiring 1.1 encounters (SD 0.4, median 1.0). Follow-up encounters were interactions done at the discretion of the nurse care manager and scheduled in collaboration with the participant after the CHAPS Assessment. These encounters, inclusive of the 6-month follow-ups, were an average of 28 min (SD 20) and varied from none to several per participant, averaging 3.3 encounters (SD 1.3, median 4.0) [23].

Participants’ had a mean age of 69.4 years (SD 10.3) and were 95.0% male. Self-identified race other than Caucasian was 23.6%. The mean Health Utilities Index 3, a measure of health-related quality of life (− 1 to 1, higher score is better), was 0.45 (SD 0.31), findings similar to a Veteran Health Administration study [47] and lower than 0.61 in another community dwelling population [48]. Among the 31 problems/topics potentially identified through the CHAPS Assessment, 74.3% of participants had Motor-related, 35.7% Cognitive, 56.4% Functional limitations, and 75.7% Falls (inclusive of risk factors) problems/topics [23].

Stakeholder responses were gathered at end of the trial. All participants were included for the evaluation of participant responses to the Notebook. A convenience sample of participants, enrolled toward the end of the trial, provided perceptions of the CHAPS intervention [8]. CHAPS nurse care managers and PD specialists surveyed for their perceptions were not considered subjects per Veterans Health Administration Institutional Review Boards, November 9, 2011.

### Data

This report used quantitative and qualitative response data gathered through either anonymous paper surveys or research assistant-administered telephone surveys. The research assistant and project manager abstracted participant Notebook perceptions, documented by CHAPS nurse care managers. All data were stored on a secure health services research server.

#### Participant surveys

The participant survey was a 17-item telephone survey about the CHAPS intervention. Thirteen survey questions addressed the Care Transition Program’s Four Pillars adapted to outpatient PD care: (1) Medication self-management, (2) Use of dynamic patient-centered record, (3) PD specialist and nurse care manager follow-up, and (4) Parkinson disease red flags. Additional questions addressed helpfulness of the nurse care manager. All items were adapted from the Care Transition Measure (CTM-15) [49] and their use in a dementia care management program [50]. Response choices were from 1 (strongly disagree) to 5 (strongly agree). Three open-ended questions elicited comments about the CHAPS Assessment (their overall impressions, what they liked best, and what they liked least) and one other elicited any additional participant comments.

#### Nurse care manager and PD specialist surveys

The CHAPS nurse care managers and PD specialists’ surveys were 14-item anonymous paper surveys about the CHAPS intervention. These were adapted from a previous program evaluation survey [50] that assessed knowledge, beliefs, and attitudes [51]. Questions were organized into five constructs: Knowledge/Understanding, Self-confidence, Clinical Appropriateness, Participant Self-management Improvement, and Endorsement. Three of the 14 questions were stated in the negative to allow for response choices to be in both the “agree” and “disagree” categories to help minimize agreement bias. In presenting results, responses to these 3 questions were rescored so all responses are reported in the same direction. An open-ended question elicited comments about how CHAPS could be improved. For PD specialists, additional questions asked about awareness of the Siebens Domain Management Model in the CHAPS documentation. If they responded “yes,” then they were asked if they felt it was a helpful way to organize participants’ problems/issues (yes, no, unsure). Also, PD specialists were asked if the participants brought Notebooks to their appointments (yes, no, unsure).

Usability - how a concept or care tool fits a particular purpose [52] - of the Siebens Domain Management Model and the Notebook was obtained from CHAPS nurse care managers. Two usability surveys, administered via telephone by a research assistant, were adapted from a web accessibility survey [53] that had been pilot tested during the development of the CHAPS Assessment.

### Analyses

SAS 9.4 statistical analysis software (SAS Institute, Inc., Cary, North Carolina) was used to provide descriptive summary statistics (i.e., frequencies and percentages). Survey rating responses for participants, CHAPS nurse care managers, and PD specialists were reported as counts and percentages for individual items.

Open card sorting was used for grouping free text comments from participants about the Notebook and comments provided by stakeholders in the surveys. Two researchers (KIC, HCS) together examined comments for word similarities (generalizations in semantics, analogies, and metaphors), distilled them into items, and sorted these items into groups that were not pre-specified. They used their knowledge of healthcare and language to refine the sorts. For items on which they disagreed, they either came to a collaborative decision or placed the item into an “Other” category. Finally, they created names for themes and attributes of related items [54, 55].

## Results

### Participant survey responses

All 28 (100%) participants agreed to take part in the 17-item survey. Overall, participants indicated highly they could self-manage medications (Pillar 1). Of the participants who recalled the three self-care tools (Notebook, CHAPS Assessment, My Action Plan) (Pillar 2), responses about usefulness varied. Participants rated highly knowing when to follow-up with the PD specialist (Pillar 3) and awareness of PD red flags (Pillar 4). Of participants who recalled speaking with CHAPS nurse care managers, responses indicated their helpfulness. Participants noted being able to talk to them and getting help in safety, activities, and self-care (Table 1).

**Table 1:** Participant responses to telephone survey about CHAPS (*n* = 28)

Pillars and Specific Questions	Strongly Disagree	Disagree n (%)	Not Sure n (%)	Agree n (%)	Strongly Agree n (%)	Don’t recall n (%)
Medication Self-management (*Pillar 1*^a^)						
I know how to take my PD medications.	–	1 (3.6)	–	24 (85.7)	3 (10.7)	–
I know what my PD medications are for.	–	1 (3.6)	2 (7.1)	23 (82.1)	2 (7.1)	–
I know the side effects of my PD medications^b^.	–	2 (7.7)	3 (11.5)	20 (76.9)	1 (3.8)	–
Use of Dynamic Patient-centered Record (*Pillar 2*)						
The Siebens Health Care Notebook that the CHAPS Nurse Care Manager mailed to me, helps my doctors, team, and me to take better care of myself.	–	9 (32.1)	7 (25.0)	8 (28.6)	2 (7.1)	2 (7.1)
I felt my CHAPS Assessment Summary, located in the back of my personal health care notebook, was useful.	–	5 (17.9)	8 (28.6)	4 (14.3)	1 (3.6)	10 (35.7)
I felt My Action Plan, located in my personal health notebook, was useful.	–	4 (14.3)	10 (35.7)	3 (10.7)	–	11 (39.3)
Parkinson disease Specialist Follow-up (*Pillar 3*)						
I know when to follow up with my PD doctor.	–	2 (7.1)	2 (7.1)	24 (85.7)	–	–
Parkinson disease Red Flags *(Pillar 4)*						
I know symptoms I should watch for to monitor my Parkinson disease (PD) condition.	–	1 (3.6)	2 (7.1)	23 (82.1)	2 (7.1)	–
I know what I should do if my PD symptoms get worse.	–	2 (7.1)	1 (3.6)	24 (85.7)	1 (3.6)	–
Additional questions addressing the Helpfulness of Nurse Care Manager						
I felt I could talk to my CHAPS Nurse Care Manager about my condition.	–	2 (7.1)	3 (10.7)	19 (67.9)	3 (10.7)	1 (3.6)
The CHAPS Nurse Care Manager helped me be as safe and active as I can be.	–	1 (3.6)	5 (17.9)	17 (60.7)	3 (10.7)	2 (7.1)
I felt my CHAPS Nurse Care Manager helped me manage my PD.	–	2 (7.1)	6 (21.4)	17 (60.7)	2 (7.1)	1 (3.6)
I felt my CHAPS Nurse Care Manager helped me manage my health overall.	–	3 (10.7)	4 (14.3)	18 (64.3)	2 (7.1)	1 (3.6)

*CHAPS* Care Coordination for Health Promotion and Activities in Parkinson’s Disease

*PD* Parkinson’s disease

^a^ The four pillar names are adapted from the Care Transition Program, Coleman E, J Am Geriatr Soc 2004; 52:1817

^b^ 2 responses were missing

A total of 20 participants provided qualitative responses (*n* = 32) about their overall impressions of the CHAPS Assessment. Three themes emerged: (1) Benefit to the Assessment (e.g., helpful/appreciative, covered all the issues, impressed, pleased with advice, and opportunity to reflect on PD); (2) Nature of interaction (e.g., caring, pleased with interaction, and confused by some assessment questions); and (3) Information gathering (e.g., comprehensive is good, providers need data). Four general observations were: recommend services, education is needed, knowledge needed early on that PD is complex, and tracking symptoms and treatment results are important.

### Participant feedback about the Notebook

Of the 140 participants who received Notebooks, 21 received no follow-up nurse care manager contact. A total of 67 (59.8%) participants had qualitative feedback documented on the Notebook. Three themes emerged: Notebook assets (*n* = 97 items), Deferring Notebook review (*n* = 28 items), and Reasons not using Notebook (*n* = 19 items) (Table 2). Additionally, participants reported care partner responses to the Notebook: Impressed (*n* = 4, e.g., very happy to have it) and Helpful/organized (*n* = 4, e.g., can take it and go).

**Table 2:** Participant perceptions of the Siebens Health Care Notebook

Themes	Attributes	Items (n)
Notebook assets (*n* = 97)	Notebook benefits^a^ (*n* = 42)	Helpful (10) Informative (8) Tool for organizing (7) Very useful (6) Personalized (5) Communicating with providers (3) Easy format to read (2) Doctor looked through it (1)
	Notebook features liked (*n* = 37)	Education sheets (10) Medication list (6) Organized by sections (6) Doctor visit sheet (5) CHAPS Assessment (3) A lot of information (2) Action tracking (2) Serves as a review (1) My Action Plan (1) Business card holder (1)
	Affirmative feelings (*n* = 18)	Impressed (5) Likes (4) Appreciative (3) Nice (3) Looking forward to using (2) Notebook-type person (1)
Reasons for participants deferring Notebook review (*n* = 28)	No time to review (*n* = 17)	
	Participant preference (*n* = 6)	Wants to review later (3) Currently not using the Notebook (3)
	Barriers to reviewing (*n* = 5)	Vision too poor to read (1) Lost glasses (1) Lost Notebook (1) Feels depressed (1) Participant appears disorganized (1)
Reasons for not using Notebook (*n* = 19)	Participant preference (*n* = 12)	Not interested (6) Can already remember everything (2) Do not need anything (2) Too busy (2)
	Notebook characteristics (*n* = 7)	Cumbersome (4) Requires writing (2) Dislikes print material (1)

^a^Benefits – defined as fulfillment of needs

### Nurse care manager survey responses

Seven of eight CHAPS nurse care managers (one unavailable) provided survey responses. Regarding the construct Knowledge/Understanding, nurse care manager responses uniformly indicated the CHAPS Assessment and Program facilitated their care of patients. They had mixed ratings relating to the influence of CHAPS on their Self-Confidence. Responses affirmed the Clinical Appropriateness in CHAPS. On Participant’s Self-Management Improvement, over half of the nurse care manager responses reflected improvements (Table 3).

**Table 3:** Nurse care manager responses to survey about CHAPS (*n* = 7)

Constructs and Specific Questions	Strongly disagree n (%)	Disagree n (%)	Undecided /neutral n (%)	Agree n (%)	Strongly agree n (%)
Knowledge/Understanding					
CHAPS Assessments, administered by CHAPS Nurse Care Managers, have provided information that will improve how I take care of my patients with Parkinson’s disease (PD). (*Knowledge (about care)*)	–	–	1 (14.3)	2 (28.6)	4 (57.1)
CHAPS Assessments have taught me something that I can use in my care of other patients with chronic disease. (*Knowledge (about care)*)	–	–	–	5 (71.4)	2 (28.6)
CHAPS Assessments have provided me with information that is relevant to the care of my patients with PD. (*Belief (relevance)*)	–	–	–	3 (42.9)	4 (57.1)
The CHAPS Program provides recommendations that are useful to help me care for my patients with PD. (*Belief (usefulness)*)	–	–	1 (14.3)	2 (28.6)	4 (57.1)
Self-Confidence					
The CHAPS Program has impacted my degree of confidence with clinical deci-sions involving Parkinson’s disease care. (*Confidence (in self)*)	–	–	3 (42.9)	1 (14.3)	3 (42.9)
Clinical Appropriateness					
CHAPS Assessments have provided diagnostic information that I agree with. (*Belief (agreement)*)	–	–	1 (14.3)	3 (42.9)	3 (42.9)
CHAPS Assessments have recommended care suggestions that I agree with. (*Belief (agreement)*)	–	1 (14.3)	1 (14.3)	2 (28.6)	3 (42.9)
The CHAPS Assessment and CHAPS Nurse Care Managers pay attention to detail and are thorough. (*Belief (thoroughness)*)	–	1 (14.3)	–	3 (42.9)	3 (42.9)
Participant’s Self-Management Improvement					
My patient(s) now have a better understanding of their medication regimen (purpose, how to take, and side effects). (*Belief (understanding)*)	–	–	2 (28.6)	3 (42.9)	2 (28.6)
My patient(s) now have a better understanding of how to manage their Parkinson’s disease. (*Belief (understanding)*)	–	–	2 (28.6)	3 (42.9)	2 (28.6)
My patient(s) now are better at following through on laboratory tests and appointments. (*Belief (follow-through)*)	–	–	3 (42.9)	3 (42.9)	1 (14.3)
My patient(s) are better able to identify signs and symptoms that indicate a risk for being admitted to the hospital. (*Belief (identifying risks)*)	–	1 (14.3)	3 (42.9)	3 (42.9)	–
Patient understands and manages his/her Siebens HealthCare Notebook. (*Belief (Notebook management)*)	–	2 (28.6)	1 (14.3)	3 (42.9)	1 (14.3)
Endorsement					
CHAPS Assessments and Nurse Care Management have encouraged me to refer my other patients with PD to the CHAPS Program^a^ (*Attitude (behavior)*)	–	1 (16.7)	1 (16.7)	2 (33.3)	2 (33.3)

*CHAPS* Care Coordination for Health Promotion and Activities in Parkinson’s Disease

*PD* Parkinson’s disease

^a^ One response was missing

Four of seven CHAPS nurse care managers provided comments (*n* = 17) to the open-ended question about how to improve CHAPS. Five themes emerged: (1) Shorten CHAPS Assessment, (2) Add care management software, (3) Provide more practice (with the Siebens Domain Management Model, huddles with PD specialists, and readiness to learn techniques), (4) Offer option of face-to-face Assessments (in clinic or via clinical video telehealth), and (5) Maintain consistent nurse care manager staffing (e.g., to build trust, facilitate collaboration, foster behavioral change, and support Notebook use). Additional comments endorsed CHAPS: positively impacted patients, supported patient-nurse care manager partnership, ideal for other enduring conditions, merits dissemination, and can incorporate future advances.

### PD specialist survey responses

A total of 10 of 12 PD specialists responded to the provider survey. Their responses indicated the CHAPS Assessment and Program facilitated patient care (see Knowledge/Understanding construct) (Table 4). Additionally, responses affirmed the Clinical Appropriateness of CHAPS, and endorsed Participant’s Self-Management Improvement. Overall, PD specialists reported they would refer their other patients to CHAPS.

**Table 4:** Parkinson disease specialist responses to survey about CHAPS (*n* = 10)

Constructs and Specific Questions	Strongly disagree n (%)	Disagree n (%)	Undecided /neutral n (%)	Agree n (%)	Strongly agree n (%)
Knowledge/Understanding					
CHAPS Assessments, administered by CHAPS Nurse Care Managers, have provided information that will improve how I take care of my patients with Parkinson’s disease (PD). (*Knowledge (about care)*)	–	1 (10.0)	1 (10.0)	5 (50.0)	3 (30.0)
CHAPS Assessments have taught me something that I can use in my care of other patients with chronic disease. (*Knowledge (about care)*)	1 (10.0)	–	2 (20.0)	4 (40.0)	3 (30.0)
CHAPS Assessments have provided me with information that is relevant to the care of my patients with PD. (*Belief (relevance)*)	–	–	1 (10.0)	5 (50.0)	4 (40.0)
The CHAPS Program provides recommendations that are useful to help me care for my patients with PD. (*Belief (usefulness)*)	–	–	4 (40.0)	1 (10.0)	5 (50.0)
Self-Confidence					
The CHAPS Program has impacted my degree of confidence with clinical decisions involving Parkinson’s disease care. (*Confidence (in self)*)	–	1 (10.0)	3 (30.0)	5 (50.0)	1 (10.0)
Clinical Appropriateness					
CHAPS Assessments have provided diagnostic information that I agree with. (*Belief (agreement)*)	–	–	2 (20.0)	5 (50.0)	3 (30.0)
CHAPS Assessments have recommended care suggestions that I agree with. (*Belief (agreement)*)	–	–	1 (10.0)	5 (50.0)	4 (40.0)
The CHAPS Assessment and CHAPS Nurse Care Managers pay attention to detail and are thorough. (*Belief (thoroughness)*)	–	–	1 (10.0)	1 (10.0)	8 (80.0)
Participant’s Self-Management Improvement					
My patient(s) now have a better understanding of their medication regimen (purpose, how to take, and side effects). (*Belief (understanding)*)	–	–	1 (10.0)	5 (50.0)	4 (40.0)
My patient(s) now have a better understanding of how to manage their Parkinson’s disease. (*Belief (understanding)*)	–	–	1 (10.0)	4 (40.0)	5 (50.0)
My patient(s) now are better at following through on laboratory tests and appointments. (*Belief (follow-through)*)	–	–	4 (40.0)	3 (30.0)	3 (30.0)
My patient(s) are better able to identify signs and symptoms that indicate a risk for being admitted to the hospital. (*Belief (identifying risk)*)	–	–	3 (30.0)	6 (60.0)	1 (10.0)
Patient understands and manages his/her Siebens HealthCare Notebook. (*Belief (Notebook management)*)	–	–	5 (50.0)	5 (50.0)	–
Endorsement					
CHAPS Assessments and Nurse Care Management have encouraged me to refer my other patients with PD to the CHAPS Program. (*Attitude (behavior)*)	–	–	1 (10.0)	2 (20.0)	7 (70.0)

*CHAPS* Care Coordination for Health Promotion and Activities in Parkinson’s Disease

*PD* Parkinson’s disease

All 10 PD specialists provided comments about CHAPS. Two themes emerged: (1) CHAPS nurse care manager/PD specialist collaboration (e.g., nurse care manager on site, reinforce consistent periodic conference telephone calls, prioritize topics for discussion, and notify PD specialist of CHAPS note availability) and (2) Helpfulness of nurse care managers (e.g., checking on patient needs, re-emphasizing clinic discussions, spending more time talking to patients than is available in clinic, facilitating completion of advance directives, coaching about long-term care decision-making, obtaining benefits and durable medical equipment, and assisting decision-making on ordering driving evaluations).

### Nurse care manager and PD specialist feedback on the Siebens domain management model

CHAPS nurse care managers (*n* = 7) provided 84 comments in the usability survey about the Siebens Domain Management Model. Open card sorting yielded two themes: Facilitators for using the model (*n* = 55 items) and Challenges in using the model (*n* = 29 items) (Table 5). Two nurse care managers stated there was nothing they disliked about the model. In rating how easy or hard the model was to understand, responses were: very easy (*n* = 2), easy (*n* = 2), easy/neutral (*n* = 1), and hard (*n* = 2). As for recommending the model to a colleague, responses were the following: yes (*n* = 3), maybe (*n* = 3), and no (*n* = 1).

**Table 5:** Usability survey themes and responses from nurse care managers (*n* = 7) about the Siebens Domain Management Model

Themes	Attributes	Items (n)
Facilitators for Using the SDMM (*n* = 55)	User-friendly (*n* = 19)	Able to put SDMM into action (6) Understandable for nurses (4) Simplifies information (4) Understandable for patient and provider (2) Helps clinicians think things through (2) Powerful tool (1)
	Person/Patient-centered (*n* = 16)	Looks comprehensively at a person’s health (4) Domains and Notebook usable by variety of patients (3) Thoughtful and detailed about the patient (3) Makes understanding a variety of patients easier (3) Reflects nursing’s holistic view (1) Helps physicians to look at whole picture (1) Patients benefit when SDMM used (1)
	Organized (*n* = 11)	Four domains are organized (4) The way 4 domains are separated (2) Good theory in organizing patients’ problems (2) Concise (1) Clear (1) Easier to categorize everything (1)
	Helpful for documentation (*n* = 9)	Good to have framework to follow (4) Helpful format for physicians and others to read (3) Formats health information understandably (2)
Challenges in Using the SDMM (*n* = 29)	Problem/topic placement (*n* = 20)	Some do not seem to fit into domains (7) Difficult to narrow things down to one domain (6) Sorting problems/topics could be easier (6) Takes time to think where certain items fit (1)
	Change is required (*n* = 9)	Documentation is different (5) Takes time to use initially (2) Why change? (2)

*SDMM* Siebens Domain Management Model (© Hilary C Siebens MD 2005)

Six of ten PD specialists were aware of the Siebens Domain Management Model, used in the CHAPS nurse care manager documentation. Four felt the model was a helpful method to organize patients’ problems, two were unsure, and none provided negative feedback.

### Nurse care manager and PD specialist feedback about the Notebook

CHAPS nurse care managers (*n* = 7) provided 74 comments in the usability survey about the Notebook. Open card sorting yielded two themes: Facilitators for coaching about the Notebook (*n* = 46 items) and Challenges to coaching about the Notebook (*n* = 28 items) (Table 6). Additionally, two nurse care managers stated there was nothing they disliked about the Notebook. For ratings of how easy or hard it was to understand the Notebook, responses were very easy (*n* = 6) and neutral (*n* = 1). As for recommending the Notebook to a colleague, responses were yes (*n* = 4), no (*n* = 2), and it depends on the patient population (*n* = 1). Two nurse care managers recommended the Notebook to others outside the study.

**Table 6:** Usability survey responses from nurse care managers about the Siebens Health Care Notebook

Themes	Attributes	Items (n)
Facilitators for coaching about the Notebook (*n* = 46)	Organized (*n* = 18)	Organized structure (8) Places for types of information (5) Concise (2) Separated into sections (2) Kept nurse care manager organized in patient care (1)
	User-friendly (*n* = 15)	Practical (6) Easy to use (4) Does what it is designed to do (2) Handy for keeping records (2) Good place to hold information (1)
	Person/Patient-Centered (*n* = 13)	Comprehensive (2) Empowering (2) Veteran-specific materials added (2) Great solution for veterans with complex health issues (2) Main communication device among providers (1) Tool for coordinating care with community providers (1) Has a specific sheet for writing questions for providers (1) Provides important information for making choices (1) Veterans can use as much or as little as they like (1)
Challenges to coaching about the Notebook (*n* = 28)	Incorporating individuals’ abilities/preferences (*n* = 14)	Everyone is different (3) Some veterans have their own ways of organizing information (3) Individualized teaching depending on veteran’s situation (2) Hard to carry for some veterans with Parkinson disease (2) More than 1–2 pages is difficult (1) Some veterans with too much on their minds (1) Some veterans love it, some do not (1) Better to teach about Notebook in person than over the phone (1)
	Notebook layout (*n* = 14)	Information could be consolidated (2) Too many sections (2) More specific to Parkinson (2) Bulky (2) Too much white (1) The location of My Action Plan (1) Pages not numbered so is hard to find contents (1) Patient’s printed medication list may be outdated (1) Not very useful (1) Old-fashioned (1)

Notebook – Siebens Health Care Notebook

Two PD specialists’ reported participants brought their Notebooks to appointments, 7 reported not seeing Notebooks, and one was not sure. Two PD specialists reported interacting with the Notebook and said something positive to participants about their use of it. Additional comments included: learned something useful from the Notebook, it helped in the care of these participants, and believed participants found it useful or helpful as did care partners.

## Discussion

Stakeholder responses to several surveys provided insights on multiple aspects of the CHAPS intervention. These surveys gave stakeholders a voice. Participants reported frequent awareness of key aspects of their PD self-care, benefits of the CHAPS Assessment, and helpfulness of CHAPS nurse care managers. PD specialists and nurse care managers noted improvements in participant self-management, especially in their medication regime and how to manage their PD. The nurse care managers acknowledged CHAPS helped in the care of their patients. PD specialists found the nurse care manager’s role helpful. Feedback on the Siebens Domain Management Model and Notebook usability identified their value and shared attributes of person/patient-centeredness, user-friendliness, and organization.

CHAPS nurse care managers’ and PD specialists’ suggestions for CHAPS likely indicated their engagement and buy-in, necessities for successful intervention dissemination [17–19]. The CHAPS Assessment could be shorter, nurse care manager availability could be more consistent than occurred during the trial, and closer collaboration could be facilitated between nurse care managers and PD specialists. Nurse care managers could be offered additional practice on the Siebens Domain Management Model (e.g., placing problems/topics in domains) and Notebook coaching (e.g., motivational interviewing, readiness to learn techniques).

Generalizability of these findings may be limited to the mostly male veteran population and the Veterans Health Administration healthcare setting. However, individuals with PD in other health settings are likely to have similar problems that could benefit from nurse care managers, guided by outpatient structured care management. Sample sizes of each stakeholder group were small; nonetheless, feedback provided rich insights. Participant knowledge and understanding about PD may have been influenced by care received outside of CHAPS, even though participants were instructed to answer survey questions in relation to their experience with CHAPS. Limited follow-up during implementation may have affected CHAPS nurse care manager perceptions of participants’ self-management.

### Implications

The nurse care manager role, as defined in the CHAPS Program, may help health care organizations improve their care quality of individuals with PD. First, CHAPS is one means for overcoming the shortage of PD nurse specialists, which is akin to initiatives like ParkinsonNet that focus on improving professionals’ education and training [2]. Second, as participants reported CHAPS nurse care managers were helpful, these interactions may contribute to care quality comparable to findings in our dementia care management study [56]. Third, nurse care managers, using the proactive standardized CHAPS components, may reduce health care organizations’ PD practice variation. The nurse care manager would be the central point person, supporting individuals with PD, care partners, and providers, especially PD specialists.

The Siebens Domain Management Model offered a beneficial organizing framework across sites and among CHAPS nurse care managers. The model allowed flexibility that honored nurse care managers’ judgment in prioritizing problems/topics in partnership with patients, facilitated problem/topic follow-up and documentation, and likely assisted with more succinct and effective communication with physicians. This demonstrated the model’s use: (1) by nurses, adding to its use by other disciplines*,* (2) in the outpatient setting, adding to inpatient findings and, thus, across the care continuum, (3) in care of individuals with PD, an enduring medical condition, adding to other diagnoses already studied, and (4) as Section Headings of the self-care Notebook (Fig. 1) [23, 26, 31, 36]. Participant and nurse care manager comments about the Notebook supported the value and role for this paper-based self-management tool. It may be used alone or to augment electronic patient portals (e.g., My HealtheVet) [45, 57, 58]. Additionally, the Notebook may assist with care continuity, as in care transitions [59], within or outside health care systems [60, 61].

Efficiency in CHAPS care delivery could improve through integrating user-friendly care management software for managing patient panels/tasks and the CHAPS Assessment with its algorithms for problem identification and associated severity [8]. Given that individuals with PD exhibit a range of disease severity, appropriate panel size for CHAPS may vary. If comprehensive proactive assessments became routinely adopted early on in PD care management, follow-up care would be more efficient as problems would be anticipated and then prevented or managed more easily. Additionally, coronavirus (COVID-19) is a significant concern for individuals with PD as it may worsen motor-related function, urinary-related symptoms, and fatigue [62] and may impose new stressors (e.g., exacerbating social isolation, limiting exercise) [63]. Therefore, during this pandemic, CHAPS may help in care efficiency through its telehealth care management.

Given stakeholder feedback, disseminating CHAPS and its components may improve partnering across healthcare and community services, especially critical in individuals with complex medical conditions [64, 65]. CHAPS may also decrease medical risks and hassles in managing enduring health conditions [42, 66] likely achieving friendlier care and greater trust [6, 67, 68].

## Conclusions

Overall, stakeholder perceptions of the proactive nurse-led CHAPS intervention indicated its value in the care of individuals with PD. Responses about the CHAPS Assessment, Siebens Domain Management Model, and Notebook self-care tool signified their usefulness. Stakeholders’ constructive suggestions indicated their engagement in CHAPS. These findings support CHAPS dissemination and contribute to research in care management.

## Supplementary Information

**Additional file 1.** Example of CHAPS Assessment for gastro-intestinal-related problems/topics. LEGEND: Items in caps are CHAPS problem/topics with associated intervention protocols: assess further, provide information, problem solve collaboratively, clinical referral, and community and social service referral. Less severe symptoms elicited only nurse care manager to review the problem further with the patient, referring to review intervention protocol as needed. More severe symptoms also triggered referrals to health providers (e.g., Parkinson’s disease specialist) [23]. CHAPS – Care Coordination for Health Promotion and Activities in Parkinson’s Disease; MD – medical doctor or other health provider.

**Additional file 2.** Self-care Tool: My Action Plan. LEGEND: Text (*) were cues for topics to be discussed in each section. The text could be left unchanged, deleted, or expanded by the nurse care manager. This Plan was placed in each participant’s personalized Notebook and updated as appropriate [23].

## Data Availability

The datasets generated and/or analyzed during the current study are not publicly available as, by the time we deidentify data to the degree acceptable, too many key variables are taken out given that veterans can be re-identified with enough social and/or personal demographic and area information. Additionally, the small pool of intervention nurse care managers and Parkinson’s disease specialists would potentially allow tracing qualitative survey data back to them.

## Abbreviations

CHAPS: Care Coordination for Health Promotion and Activities in Parkinson’s Disease
PD: Parkinson disease
Notebook: Siebens Health Care Notebook
SD: Standard deviation
CTM-15: Care Transition Measure
SAS: Statistical analysis software
