# Supplementary material for: Stakeholder perceptions of components of a Parkinson disease care management intervention, care coordination for health promotion and activities in Parkinson’s disease (CHAPS)
Source: BMC Neurol. 2020 Dec 2;20:437. doi: 10.1186/s12883-020-02011-9 (PMC7708498; doi:10.1186/s12883-020-02011-9)
Supplement: Supplementary file 1 — Additional file 1. Example of CHAPS Assessment for gastro-intestinal-related problems/topics. LEGEND: Items in caps are CHAPS problem/topics with associated intervention protocols: assess further, provide information, problem solve collaboratively, clinical referral, and community and social service referral. Less severe symptoms elicited only nurse care manager to review the problem further with the patient, referring to review intervention protocol as needed. More severe symptoms also triggered referrals to health providers (e.g., Parkinson’s disease specialist) [23]. CHAPS – Care Coordination for Health Promotion and Activities in Parkinson’s Disease; MD – medical doctor or other health provider. [file 12883_2020_2011_MOESM1_ESM.docx]

Additional File 1 Example of CHAPS Assessment for gastro-intestinal-related problems/topics

| GASTRO-INTESTINAL-RELATED PROBLEMS/TOPICS  Patient reports:  [ ] gaining [ ] losing weight in the last 3 months (or clothes fitting differently) [ ] no change | | | | | | | | |
| --- | --- | --- | --- | --- | --- | --- | --- | --- |
| If gain or loss, then WEIGHT/NUTRITION | | | | | | | | |
| If “Yes, losing”, was participant trying to lose weight: | | [ ] Yes | | | [ ] No | | [ ] Not Sure/Deferred | |
| If No or Not Sure/Deferred, then MD Referral | | | | | | | | |
| If “Yes, gaining”, was participant trying to gain weight: | | [ ] Yes | | | [ ] No | | [ ] Not Sure/Deferred | |
| If No or Not Sure/Deferred, then MD Referral | | | | | | | | |
|  | | | | | | | | |
| The participant has experienced the following eating/nutritional problems since last seeing doctor: | | | | | | | | |
| [ ] Marked loss of appetite | | | | | | | | |
| If checked, then WEIGHT/NUTRITION and MD Referral | | | | | | | | |
| [ ] Problems chewing | | | | | | | | |
| If checked, then WEIGHT/NUTRITION and Dental Referral | | | | | | | | |
| [ ] Problems swallowing (choking w/eating and drinking or taking pills) | | | | | | | | |
| If checked, then SWALLOWING and MD Referral | | | | | | | | |
| [ ] Dental problems or pain in teeth, gums, or mouth | | | | | | | | |
| If checked, WEIGHT/NUTRITION and Dental Referral | | | | | | | | |
| [ ] Does not have enough money to buy food | | | | | | | | |
| If checked, then WEIGHT/NUTRITION and ADDRESSING FINANCIAL CONCERNS | | | | | | | | |
| [ ] Does not have access to food (e.g. no one to pick up food/groceries) | | | | | | | | |
| If checked, then WEIGHT/NUTRITION | | | | | | | | |
| STOMACH AND BOWELS | | | | | | | | |
| *(in the last seven days)* | | Not at all | Several days | | | More than half the time | | |
| Stomach felt full before meal finished | | [ ] | [ ] | | | [ ] | | |
| Nauseated or felt was going to throw up right after eating a meal | | [ ] | [ ] | | | [ ] | | |
| Felt bloated | | [ ] | [ ] | | | [ ] | | |
|  | |  |  | | |  | | |
| *(In the last month)* | | Not at all | Several days | | | More than half the time | | |
| Problems with diarrhea | | [ ] | [ ] | | | [ ] | | |
| Problems with constipation | | [ ] | [ ] | | | [ ] | | |
| Problems with bowel incontinence | | [ ] | [ ] | | | [ ] | | |
| If any of above = Several days, then GASTRO-INTESTINAL RELATED | | | | | | | | |
| If any of above = More than ½ the time, then GASTRO-INTESTINAL RELATED and MD Referral | | | | | | | | |
| SALIVA AND DROOLING | | | | | | | | |
| Participant reports problems with excess saliva: | | [ ] Yes | [ ] No | | | [ ] Not Sure/Deferred | | |
| If Yes, is it | [ ] mild, | [ ] slight, | | [ ] moderate, | | | | [ ] severe? |
| If moderate or severe, then SWALLOWING and MD Referral | | | | | | | | |
