# Supplementary material for: Stakeholder perceptions of components of a Parkinson disease care management intervention, care coordination for health promotion and activities in Parkinson’s disease (CHAPS)
Source: BMC Neurol. 2020 Dec 2;20:437. doi: 10.1186/s12883-020-02011-9 (PMC7708498; doi:10.1186/s12883-020-02011-9)
Supplement: Supplementary file 2 — Additional file 2. Self-care Tool: My Action Plan. LEGEND: Text (*) were cues for topics to be discussed in each section. The text could be left unchanged, deleted, or expanded by the nurse care manager. This Plan was placed in each participant’s personalized Notebook and updated as appropriate [23]. [file 12883_2020_2011_MOESM2_ESM.docx]

Additional File 2 Self-care Tool: My Action Plan

**Care Coordination for Health Promotion and Activities**

**In Parkinson’s Disease (CHAPS)**

**My Action Plan (Initial)**

[Participant’s Name] [Date]

REMEMBER – Use your Siebens Health Care Notebook to organize your health information. Take it with you to all your health care appointments!

HERE ARE NEXT STEPS YOU CAN DO:

* Read through your Notebook.

* Write your name inside the Notebook.

* Add any other information *if* you want to and have the time.

THE BODY, SECTION 1

* Review your medications. See the list I put in your notebook.

* Review the protein-levodopa fact sheet.

* Please review education sheet(s) I put in your Notebook.

THE MIND, SECTION 2

* Consider joining us for the Veteran Affairs Parkinson’s Disease at Home Telephone support

group/education program.

* Please review education sheet(s) I put in your Notebook.

ACTIVITIES, SECTION 3

* Keep active!

* Do what you enjoy!

* Please review education sheet(s) I put in your Notebook.

SURROUNDINGS, SECTION 4

* Please review education sheet(s) I put in your Notebook.

NEXT STEPS YOUR CARE MANAGER WILL DO:

* I will communicate a summary of our discussion to your provider(s)

*I have put a copy of your CHAPS Assessment in your Notebook for your provider(s). *Be sure to*

*show it to them; however, keep it to help coordinate your care.*

OUR NEXT PHONE CALL to review your progress and care: _________________

* Please give me feedback on identified priority problems and this tentative action plan.

­­­­­­­­­­

[Name]

Nurse Care Manager (*credentials*)

(Terms Body, Mind, Activities, Surroundings © Hilary C. Siebens 2008 used with permission.)
